# ResNet Based Deep Features and Random Forest Classifier for Diabetic Retinopathy Detection †

**DOI:** 10.3390/s21113883

**Published:** 2021-06-04

**Authors:** Muhammad Kashif Yaqoob, Syed Farooq Ali, Muhammad Bilal, Muhammad Shehzad Hanif, Ubaid M. Al-Saggaf

**Affiliations:** 1 School of Systems and Technology, University of Management and Technology, UMT Road, C-II Johar Town, Lahore 54782, Pakistan; f2018279019@umt.edu.pk (M.K.Y.); farooq.ali@umt.edu.pk (S.F.A.); 2 Center of Excellence in Intelligent Engineering Systems, Department of Electrical and Computer Engineering, King Abdulaziz University, Jeddah 21589, Saudi Arabia; mshanif@kau.edu.sa (M.S.H.); usaggaf@kau.edu.sa (U.M.A.-S.)

**Keywords:** Deep Features, ResNet-50, Random Forest, diabetic macular edema, Referable DME, Inception-v3

## Abstract

Diabetic retinopathy, an eye disease commonly afflicting diabetic patients, can result in loss of vision if prompt detection and treatment are not done in the early stages. Once the symptoms are identified, the severity level of the disease needs to be classified for prescribing the right medicine. This study proposes a deep learning-based approach, for the classification and grading of diabetic retinopathy images. The proposed approach uses the feature map of ResNet-50 and passes it to Random Forest for classification. The proposed approach is compared with five state-of-the-art approaches using two category Messidor-2 and five category EyePACS datasets. These two categories on the Messidor-2 dataset include ’No Referable Diabetic Macular Edema Grade (DME)’ and ’Referable DME’ while five categories consist of ‘Proliferative diabetic retinopathy’, ‘Severe’, ‘Moderate’, ‘Mild’, and ‘No diabetic retinopathy’. The results show that the proposed approach outperforms compared approaches and achieves an accuracy of 96% and 75.09% for these datasets, respectively. The proposed approach outperforms six existing state-of-the-art architectures, namely ResNet-50, VGG-19, Inception-v3, MobileNet, Xception, and VGG16.

## 1. Introduction

**Figure 1:**
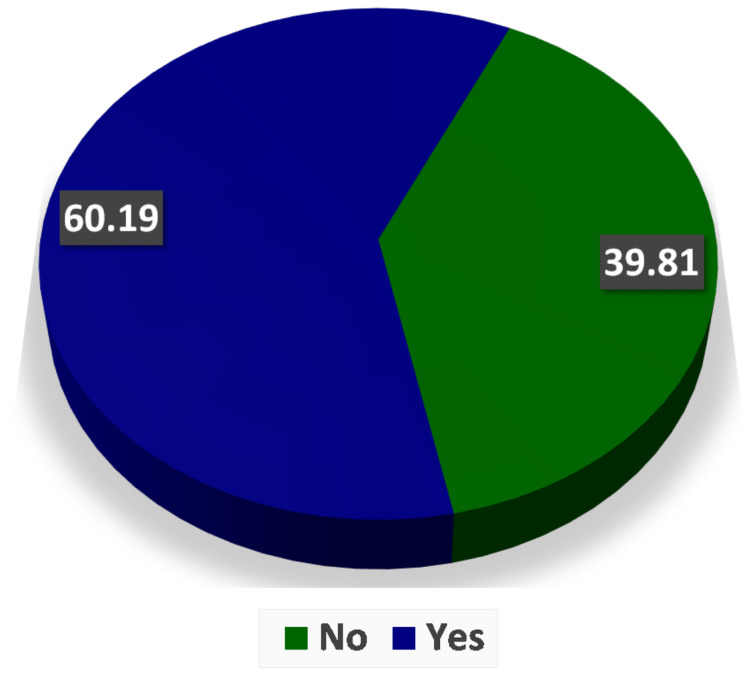
Study of 108 patients shows the prevalence of diabetic retinopathy in the Ophthalmology Clinic at a Tertiary Care Hospital, Telangana State [7].

**Figure 2:**
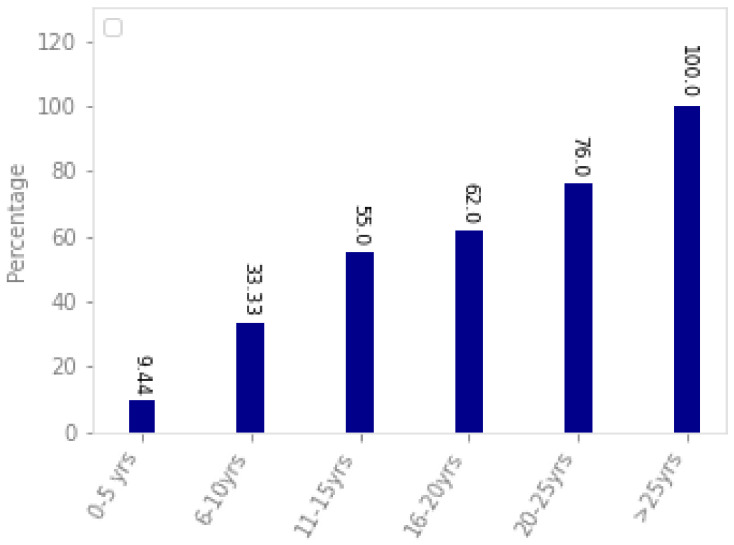
Association of diabetic retinopathy with the duration of diabetes in patients [8].

Diabetes Mellitus, or simply diabetes, is a disorder that can cause high glucose concentration in blood for a long period. It was estimated that more than 370 million people could be affected by this disease worldwide [1,2]. It was further predicted that this number will increase and become approximately 600 million by 2040 [3]. High glucose levels could damage retina blood vessels. Hence, people with diabetes of both type 1 and 2 are at a high risk of developing diabetic retinopathy [4]. Figure 1 shows the results of a study conducted in the Opthalmology Clinic at a Tertiary Care Hospital, Telangana State, India. According to this, more than 60% of patients were found to have diabetic retinopathy. The risk of getting diabetic retinopathy in individuals is 18% in India and around 28.5% in the US [5]. If left undiagnosed and untreated, diabetic retinopathy can cause blindness [6]. Most guidelines recommend the periodic screening of people depending upon the severity of diabetic retinopathy because early treatment is crucial to contain this disorder. Figure 2 shows the association of diabetic retinopathy with patients having diabetes. It can be concluded that patients having diabetes for a longer duration have greater chances of getting diabetic retinopathy. The patients having diabetes for more than 25 years have 100% chances of getting diabetic retinopathy, while this percentage reduces to 9.44 for patients having diabetes for less than five years.

The guidelines recommend that the patients with type 1 diabetes should consult an ophthalmologist or optometrist for a thorough eye examination within 3–5 years after the onset of diabetes [9]. However, detection of diabetic retinopathy can be challenging if the process involves manual evaluation by the reviewers because it is more time-consuming and can result in delayed treatment [10]. Moreover, it also requires high expertise and valuable equipment that is lacking in less privileged areas. These problems can be solved by the automated grading of diabetic retinopathy [11,12]. Various solutions have been proposed in this area where deep learning seems to provide promising results. Deep learning algorithms have been shown to outperform conventional approaches such as fuzzy techniques, morphological operations, random forest classifier, etc. They, however, require high computational power and a large dataset repository to generate better results.

Deep learning, a branch of machine learning, has shown promising results in recent years [13]. In 2014 and 2015, the performance of GoogLeNet and ResNet surpassed the human accuracy of image recognition [13]. In the current era, the easy availability of increased computing power coupled with high graphical processing capability and availability of large datasets created more space for the implementation of deep learning algorithms [14].

Deep learning approaches have been highly competitive in a large number of tasks of computer vision and image analysis, significantly exceeding all the classical image analysis techniques [15,16]. Several deep-learning algorithms have been developed to analyze retinal fundus images to construct automated computer-aided algorithms that have applications in various areas. One of the areas where we can apply these algorithms is the detection of various eye diseases, specifically diabetic retinopathy.

This paper, an extension of our published work [17], made the following contributions to the body of knowledge.

- The proposed approach for the detection and grading of diabetic retinopathy uses the deep features of a fine-tuned ResNet-50 that are extracted from its pooling layer. The classification is performed using the Random Forest (RF) classifier contrary to the traditional scheme of using the fully connected layer.
- The proposed scheme for feature extraction and classification outperforms existing deep architectures (ResNet-50, VGG-19, Inception-v3, MobileNet, Xception, and VGG16) in terms of execution time and classification accuracy on EyePACS and Messidor-2 datasets for detection and grading of diabetic retinopathy.
- The proposed approach exhibits better results than the existing techniques for the detection and grading of diabetic retinopathy on the above-mentioned two datasets.

This study is summarized as below. Related works are presented in Section 2, while datasets are illustrated in Section 3. Section 4 illustrates the proposed architecture. Experiments and results are presented in Section 5. Conclusions and future works are mentioned in Section 6.

## 2. Related Work

In 2016, Gulshan et al. proposed an algorithm based on Inception-V3 to detect diabetic retinopathy using EyePACS and Messidor-2 data set containing 9963 and 1748 images, respectively [5]. Their approach gave 98.1% specificity and 90.3% sensitivity for the EyePACS dataset, with 87% sensitivity and 98.5% specificity for the Messidor-2 dataset. To speed up their approach, they used batch normalization and pre-initialized weights from the ImageNet dataset. In the same year, Pratt et al. used data augmentation in their CNN-based architecture using a Kaggle based dataset to diagnose diabetic retinopathy [18]. They trained on 80,000 images using high-end GPU and obtained 95% sensitivity and 75% validation accuracy. Their accuracy was reduced when they extended their proposed algorithm from two to five categories i.e., normal, mild, moderate, severe, and proliferative.

In 2017, Quellec et al. proposed an algorithm to detect diabetic retinopathy by creating heatmaps using ConvNet [19]. Using 90,000 images of the Kaggle based dataset and 110,000 images of the e-optha dataset, they achieved 95.4% and 94.9% accuracy, respectively. In the same year, Akiba et al. set up an experiment consisting of 90 epochs of ResNet on 1024 GPUs using the ImageNet dataset [20]. They were able to train the ImageNet dataset in 15 min with an accuracy of 74.9% with a large mini-batch size in parallel training.

In 2017, Abbas et al. proposed an approach based on deep visual features extracted using techniques of gradient location-oriented histogram techniques for grading of diabetic retinopathy into five categories [21]. They achieved an accuracy of 92.4% on 750 images. In 2017, Mansour et al. applied AlexNet with multiple optimization techniques for computer-aided diagnosis of diabetic retinopathy [22]. On the Kaggle dataset, this CNN architecture exhibited a classification accuracy of 95.26% with principal component analysis and 97.93% with FC7 features.

Gosh et al. implemented their CNN-based model on a dataset of 30,000 images, achieving an accuracy of 95% and 85% for two and five category problems of diabetic retinopathy, respectively [23]. As a pre-processing, they applied denoising techniques. In 2017, Ardiyanto et al. proposed a compact algorithm Deep-DR-Net that can be loaded in small embedded boards [24]. Their deep learning system was said to enable future low-cost embedded systems that can detect disease with high performance. In the same year, Takahashi et al. proposed a deep learning algorithm for the grading of diabetic retinopathy by modifying GoogLeNet [13]. In the grading, they obtained 81%, while, in real prognosis, they achieved 96% accuracy on the Jichi Medical University data set with 9939 samples. They said their system can also be applied to other diseases for improved prognosis. Dutta et al. proposed a deep neural network for the detection of diabetic retinopathy [25]. To identify class thresholds, they used the Fuzzy C-means algorithm. They achieved 82.3% accuracy on a Kaggle data set with over 35,000 images. Yu et al. proposed a CNN with 16 layers for exudate detection, an essential task for the detection of diabetic retinopathy [26]. They gave a local region with dimensions 64 × 64 as an input to their CNN and obtained 88.85% sensitivity and 96% specificity on Fundus images.

Yang et al. proposed a two-staged deep CNN for the analysis of diabetic retinopathy [27]. Their algorithm pointed out the type of lesions with their location in fundus images while identifying the severity grade in each image. By the introduction of an unbalanced weighting map, the performance of their proposed algorithm was further improved. In the EyePACS dataset, they labeled 12,206 lesion patches and re-annotated the grades of 23,595 images. The accuracy of their proposed algorithm was 95.95%.

In 2017, Kanungo et al. used Inception-v3 architecture for automated detection of diabetic retinopathy on the Kaggle dataset and California Health Care Foundation (CHCF) dataset [28]. They achieved an accuracy of 82% and 88% for a batch size of 64 and 128, respectively. In the same year, Masood et al. used transfer learning on CNN based on Inception-V3, which was pre-trained on ImageNet [29]. On the five-category dataset of EyePACS, they were able to achieve 48.2% accuracy.

Kwasigroch et al. worked on 88,000 images of the EyePACS dataset for the detection of diabetic retinopathy and proposed a class coding technique related to predicted score and target score [30]. Their VGG-D architecture achieved 51% in the assessing stage and 82% in detecting diabetic retinopathy. In the same year, Wang et al. used Inception-V3 to detect diabetic retinopathy to demonstrate its effectiveness on the EyePACS dataset [1]. They tested on AlexNet, VGG16, and Inception-V3 and obtained 37.43%, 50.03%, and 63.23% accuracy, respectively. Garcıa et al., in the same year, worked on 35,126 images of the EyePACS dataset on CNN-based architecture [31]. They achieved a validation accuracy of 83.68% with 93.65% specificity by eliminating noise, performing normalization, and using various hyperparameters. See Table 1 for the related work.

In 2018, Rajalakshmi et al. worked on a database graded by International clinical diabetic retinopathy generated through smart-phone based devices [35], which had 296 patients. Their AI-based algorithm EyeArt achieved 80.2% specificity and 95.8% sensitivity which showed that an AI-based system can be used for mass screening of diabetic patients. In the same year, Wan et al. demonstrated the capability of different CNN models in identifying diabetic retinopathy, namely AlexNet, ResNet, GoogLeNet, and VGGNet after applying transfer learning and hyper-parameter tuning [32]. Using a dataset of 35,126 images from the EyePACS website, they were able to achieve 89.75%, 95.68%, 93.17%, 93.73%, 93.36%, and 90.40% accuracy for AlexNet, VGGNet-s, VGGNet-16, VGGNet-19, GoogLeNet, and ResNet, respectively. In the same year, Poplin et al. identified risk factors for a different group of sample categories including age, gender, smoking status, cardiac event history, and blood pressure using retinal fundus images trained using deep learning network [36]. These risk factors could indicate possible diabetic retinopathy patients in advance. They trained their network on a sample of 284,335 patients and validated their model on two different datasets with 12,026 and 999 samples. They showed how deep learning can predict this new knowledge from fundus image samples using the UK Biobank dataset and EyePACS dataset.

In 2019, Raumviboonsuk et al. compared how the deep learning algorithm Inception-v4 performed against human graders in Thailand [37]. In addition, 25,326 images were used for the experiment which showed that human graders had an accuracy of 78%, while the deep learning approach achieved 85% which showed that deep learning can be used as a valuable tool in disease detection. Moreover, deep learning reduced the false-negative rate by 23% but slightly increased the false-positive rate to 2%.

In 2019, Zhang et al. proposed a deep diabetic retinopathy system for grading and identification of diabetic retinopathy which was based on a combination of customized deep neural networks [38]. This system used ensemble learning and transfer learning for the detection of severity from images. They used a dataset of 13,767 images from 1872 patients collected from endocrinology, ophthalmology, and physical examination centers. The model achieved a specificity of 98.9% and sensitivity of 98.1%. In the same year, Sahlsten et al. provided novel results on the dataset of 41,122 graded images of 14,624 patients taken from Digifundus Ltd in Finland [39]. Their proposed architecture was based on Inception-V3 architecture which was pre-trained on the ImageNet dataset. Their model had an accuracy of 98.7%.

In 2019, Qummar et al. proposed a model based on an ensemble of five CNN models including Dense-169, Xception, Dense-121, ResNet-50, and Inception-v3 for the classification of different severity levels of diabetic retinopathy [6]. Their model had precision of 84%, 51%, 65%, 48%, and 69% for class 0, 1, 2, 3, and 4, respectively, on the EyePACS dataset. In the same year, Shanthi et al. proposed Alexnet based architecture using suitable rectified linear activation Unit, pooling, and softmax layers to classify the severity level of diabetic retinopathy [33]. After validating their algorithm on the Messidor dataset, they were able to achieve 96.6%, 96.2%, 95.6%, and 96.6% accuracy for healthy, stage 1, stage 2, and stage 3 cases of diabetic retinopathy, respectively.

In 2020, Shankar et al. proposed the HPTI-v4 model based on Inception-V4 for the detection of diabetic retinopathy [4]. In the pre-processing, they improved the contrast of images by the contrast limited adaptive histogram equalization technique. For the segmentation of images, they used a histogram-based segmentation model. They used their HPTI-v4 model for feature extraction and multi-layer perceptron for classification. During the assessment of their model on the Messidor dataset, they achieved 99.49% accuracy. Shankar et al., in the same year, proposed a deep learning-based SDL model for the classification of diabetic retinopathy and achieved an accuracy of 99.28% on the Messidor dataset [34]. As a pre-processing step, they denoised the edges of images and then applied histogram-based segmentation to extract useful regions.

A summary of the above discussion has been given in Table 1, highlighting the respective performances of various techniques on standard datasets. It should be noted, however, that each reported work has used a different subset of the whole dataset discarding examples with poor image quality. This makes it difficult to fairly compare all the reported works. In our experimentation, we have used all the images in the standard datasets and reproduced results using different architectures ourselves. Another difficulty in evaluating various approaches is that different works have reported their respective results using different metrics such as sensitivity, specificity, or accuracy, etc. In this work, we have chosen accuracy as the evaluation metric to be consistent with the most recent significant works reported in Zago [40], Gar [41], Orlando [42], Voets [43], and Carr [44].

## 3. Dataset

A standard, publicly accessible dataset provided by ADCIS is the Messidor-2 (http:/www.adcis.net/en/third-party/messidor2/, accessed on 1 June 2020) [45,46] that contains 1748 photographs and 874 test subjects. In Table 2, two categories are labeled for the dataset, namely 1 for ‘Referable DME’ and 0 for ‘No Referable DME’.

On the Kaggle competition website, the EyePACS dataset (https:/www.kaggle.com/c/diabetic-retinopathy-detection/data, accessed on 1 June 2020) [5] is publicly accessible and contains 35,126 images. The California Healthcare Foundation and EyePACS met Kaggle’s competition with their image repository having a varied degree of disease with their confidence in artificial intelligence. Each image is annotated with the identification number of the left or right eye and the subject Id. In Table 3, five categories are labeled for the dataset, namely 4 for ‘Proliferative Diabetic Retinopathy’, 3 for ‘Severe’, 2 for ‘Moderate’, 1 for Mild, and 0 for ‘No Diabetic Retinopathy.

**Figure 3:**
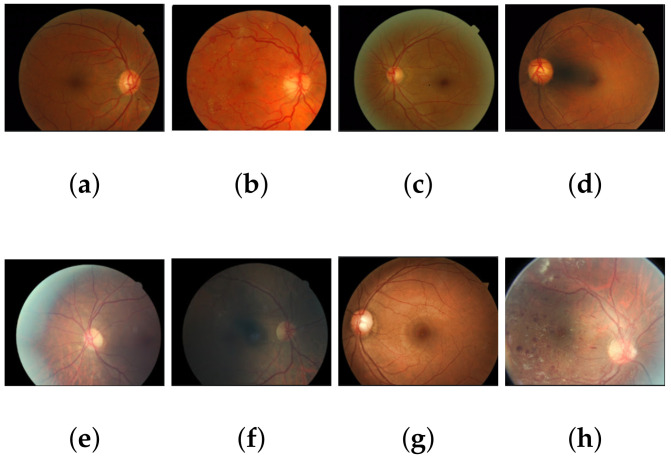
Key frames of Messidor-2 (**a**–**d**) dataset and EyePACS dataset (**e**–**h**) depicting their relative alignment and noise pattern.

Figure 3 shows the key images of Messidor-2 and EyePACS data having varying grades of diabetic retinopathy. As can be noticed from these sample images, Messidor-2 generally contains images aligned across with retina outlines. EyePACS examples, on the other hand, do not conform to this alignment. Moreover, EyePACS images contain more noise and other artifacts as well. This makes EyePACS a more challenging dataset as compared to Messidor-2 as discussed in Section 5.

## 4. Methodology

**Figure 4:**
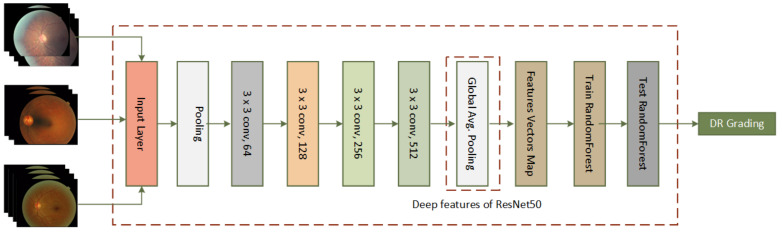
The proposed architecture using deep features of ResNet-50 in combination with a Random Forest classifier.

The proposed approach uses deep features of ResNet-50 along with Random Forest as a classifier for the detection and grading of diabetic retinopathy. High-level features obtained from the average pooling layer of trained ResNet-50 are fed to a random forest classifier as shown in Figure 4. This figure also shows the major layers of ResNet-50, namely: 3 × 3 conv 64, 3 × 3 conv 128, 3 × 3 conv 256, 3 × 3 conv 512, feature vector map, etc.

The depth of the deep network plays a pivotal role in their performance. With the increase in layers, the model gives better performance. However, it has also been observed that the addition of layers may increase the error rate. This is named as an issue of vanishing gradients. The residual neural network, also known as ResNet, was introduced to address this problem [47].

**Figure 5:**
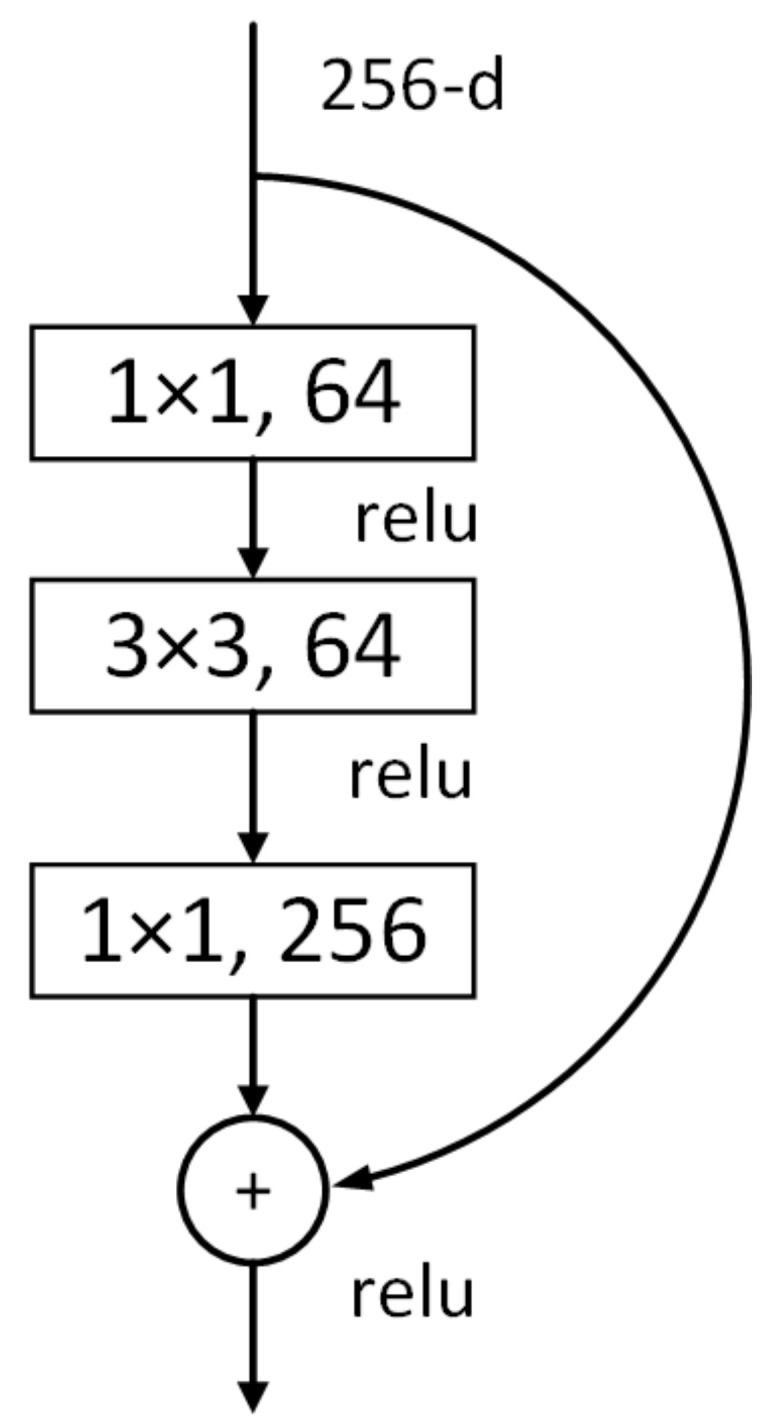
Building block of Residual Network depicting ReLu activation function and various convolution layers [47].

Figure 5 shows the building block of a residual network showing ReLu activation function and various convolution layers (1 × 1 64, 3 × 3 64, 1 × 1 256) [47]. Residual Network uses the skip connection to indiscriminately allow some input to the layer to incorporate the flow of information and also to prevent its loss, hence, addressing the problem of vanishing gradients (which also suppresses the generation of some noise). Suppressing the noise means averaging the models, which keeps a balance between precision and generalization. To achieve higher precision and an estimated level of traversal, the most efficient way is to increase more labeled data. The structure of ResNet speeds up the training of ultra-deep neural networks and increases the model’s accuracy on large training data:

(1) H(x)=F(x)+x

where:

*x* = shows the input of building block.

*F(x)* = shows the output of the layer within the building block of the residual network. 

After the training of the residual network with 20 epochs, the features were extracted from their average global pooling layer. These features were most detailed and unique as this model averaged out all the activations of the final convolution layer. Due to parameter limitations, the global average pooling does not require optimization. Moreover, owing to spatial translation, it is more robust to the input as it summarizes spatial information. The dropout was set to 0.2 to reduce the overfitting. The input layer was changed to 4 × 4 × 2048 after a series of convolution operations in the residual block, and the global average pooling layer changed the feature’s shape to 1 × 1 × 2048 with 9:1 train and validation samples. We used these features to establish a final diagnosis of the image via a second-level random forest classification model.

The Random Forest classifier has a significant effect on the recognition of diabetic retinopathy due to its ability to process large features even with small sample size. It is an ensemble classifier that can train many decision trees in parallel by a combination of classification, bagging, and regression tree. We used the Scikit-learn library that uses the Gini Importance equation to calculate the importance of each decision tree in a random forest as shown in Equation (2):

(2) $$mi_{j}=w_{j}C_{j}-W_{left(j)}C_{left(j)}-W_{right(j)}C_{right(j)}$$

where:

$mi_{j}$ = importance of node *j*

$C_{j}$ = the impurity value of node *j*

$w_{j}$= weighted samples reaching node *j*

$W_{right(j)}$ = weighted samples on child node from right split on node *j*

$W_{left(j)}$ = weighted samples on child node from left split on node *j*

$C_{right(j)}$ = the impurity value on child node from right split on node *j*

$C_{left(j)}$ = the impurity value on child node from left split on node *j*

The feature importance of the function was measured as the decrease in node impurity weighted by the likelihood of reaching that node. The higher node probability values demonstrated the significance of a function. It is possible to measure the node likelihood by the number of samples hitting the node, divided by the total number of samples.

In the feature importance method, the Scikit-learn obtained final feature importance by taking the feature importance of each tree and dividing it by the total number of decision trees as shown in Equation (3):

(3) $$RFfi_{i}=\frac{\sum_{j\in alltrees}normfi_{ij}}{T}$$

where:

$normfi_{ij}$ = the feature importance of the normalized function for *I* in tree *j*

$RFfi_{i}$ = the feature importance of the function determined from all trees in the model of the Random Forest

*T* = total amount of trees 

The final feature importance of the function, at the level of the Random Forest, was its average over all the trees. On each tree, the sum of the significant value of the function was determined and divided by the total number of trees. Final results from the random forest were taken for comparative analysis on the performance of other models for diabetic retinopathy detection. In the Random Forest, the parameters used were ‘criterion’ = entropy, ‘min-samples-leaf’ = 1, ‘min-samples-split’ = 2 and ‘random-state’ = 1. These parameters gave the best accuracy for both datasets.

## 5. Experiments and Results

The comparison was made with the proposed approach and state-of-the-art architectures, namely: ResNet-50, VGG-19, Inception-v3, MobileNet, Xception, and VGG16 [47,48,49,50,51,52]. The two datasets including EyePACS and Messidor-2 were used in this comparison.

### 5.1. Environment

Google Colab was used in the experimentation that offers free TPU and GPU on the cloud. The GPU acceleration of NVIDIA Tesla was used due to the high computational nature of the experiments. Using the Colab interface, the datasets were first downloaded directly to the Google Drive and then executed using Python programming language. Both datasets were pre-processed and resized (128 × 128 × 3). All the experimental results given in this section can be replicated through the provided open-source code available at the link given in Supplementary Materials.

### 5.2. Experiment 1: Messidor

Table 4 shows that the proposed approach exhibited better percentage accuracy as compared to existing architecture on the Messidor-2 dataset with two categories. The proposed approach uses a Random Forest classifier in place of the ResNet-50 classifier. The Random Forest classifier can process large features even with a smaller number of samples. This results in an increase in accuracy from 81.99% to 96%. The proposed approach gives even better results than VGG16, which uses 138 million trainable parameters in comparison with only 23 million for the deep features extracted from ResNet-50. This clearly shows that the proposed approach of using a Random Forest classifier in place of a ResNet-50s conventional fully connected layer greatly enhances its discrimination power.

Table 5 compares Zago [40], Gar [41], Orlando [42], Voets [43], and Carr [44] with the proposed approach on the Messidor-2 dataset.

### 5.3. Experiment 2: EyePACS

Our proposed approach showed improved percentage accuracy as compared to existing architectures using the EyePACS dataset with five categories as shown in Table 4. The proposed approach uses a Random Forest that typically deals well with high-dimensional data [53].

It can also be observed that accuracy on all the approaches gives a lesser percentage accuracy on EyePACS as compared to the Messidor-2 dataset [47]. The reason includes a large number of raw and noisy images. The images of the EyePACS dataset contain high-resolution retina images taken under a variety of imaging conditions. Moreover, the left and right fields are provided for every subject. The images come from different types of cameras and models that can affect visual appearance. In some images, the macula is on the left while the optic nerve is on the right for the right eye. Other images look inverted, as one sees in a typical live eye exam. As the data are created in an uncontrolled real-world environment, they contain lots of noise including artifacts, being out of focus, overexposed, or underexposed.

Table 6 shows that the proposed approach outperformed Suriyal [54], Kaj [55], Mas [29], and Wang [1], while performing competitively with Pratt [18] in terms of accuracy using EyePACS datasets.

### 5.4. Experiment 3: Execution Time

**Figure 6:**
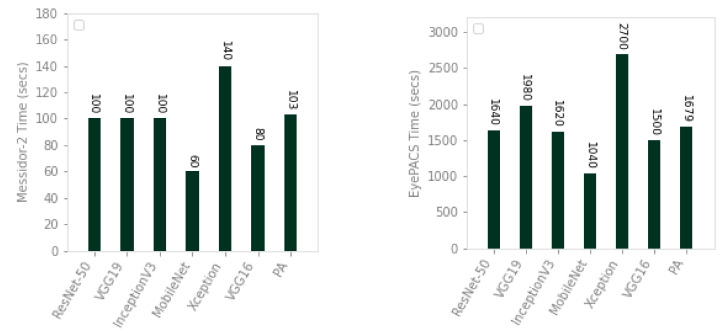
Comparison of existing approaches and proposed approach in terms of execution time (training and testing) on Messidor-2 (**left**) and EyePACS (**right**) datasets using 10-fold cross validation.

We compared the proposed approach with ResNet-50, VGG19, Inception-v3, MobileNet, Xception, and VGG16 in terms of time. The results have been shown in Figure 6. The time for existing and proposed approaches was calculated using GPU accelerated run time of Google Colab, in each experiment, which was randomly assigned from their inventory of Nvidia K80s, T4s, P4s, and P100s. For consistency of resources, we connected our run time to a GPU and performed tests on the same connection. The proposed approach is 1.35 times faster than Xception on Messidor-2, while it is 1.17, 1.60 times faster than VGG19 and Xception on EyePACS. As compared to the existing deep architectures, our proposed approach achieves greater accuracy with comparable time efficiency.

## 6. Conclusions and Future Work

In this paper, we proposed a deep learning-based approach, for the classification and grading of diabetic retinopathy. The proposed approach was compared with six state-of-the-art approaches and yielded better results. The proposed approach achieved an accuracy of 96% on the Messidor-2 dataset (two categories) including ‘Referable DME’ and ‘No Referable DME’. It obtained 75.09% accuracy on the EyePACS dataset with five classes, namely: ‘Proliferative diabetic retinopathy’, ‘Severe’, ‘Moderate’, ‘Mild’, and ‘No diabetic retinopathy’. The development of hand-crafted features could become challenging due to different lighting conditions, noise, and the presence of artifacts in images. The feature extraction learned from the data due to convolutional layer abilities seems to generate more promising results.

In the future, we aim to extend our proposed architecture to work on the real-world unfiltered images in real-time. For clinical applications, more testing is required on real scenarios and the system should be made to be more robust. Such systems could assist health practitioners with consulting more patients due to their fast diagnoses. The accuracy decreases from 96%, on a two category Messidor-2 dataset, to 75.09% on a five category EyePACS dataset because of the curse of dimensionality. Therefore, the addition of large image repositories for deep learning solutions will be in high demand in the future.

## Figures and Tables

**Table 1:** Summary of related work.

Ref.	Proposed	Result	Dataset
2016, Gulshan et al. [5]	Algorithm based on Inception-V3	98.1% specificity, 90.3% sensitivity for EyePacs and 87% sensitivity, 98.5% specificity for the Messidor-2	EyePACS, Messidor-2
2016, Pratt et al. [18]	Algorithm to detect diabetic retinopathy by creating heatmaps using ConvNet	95% sensitivity and 75% validation	EyePACS
2017, Quellec et al. [19]	Data augmentation on CNN-based architecture	95.4% on EyePACS and 94.9% on e-optha	EyePACS, E-Ophtha
2017, Mansour et al. [22]	AlexNet with multiple optimization techniques	Accuracy of 95.26% with principal component analysis and 97.93% with FC7 features	EyePACS
2017, Gosh et al. [23]	CNN-based model with denoising techniques	Accuracy of 95% and 85% for two and five category problems, respectively	EyePACS
2017, Dutta et al. [25]	Deep neural network with Fuzzy C-means algorithm	82.3% accuracy	EyePACS
2017, Yang et al. [27]	Two-staged deep CNN with the introduction of an unbalanced weighting map	95.95% accuracy	EyePACS
2017, Kanungo et al. [28]	Inception-v3 architecture	Accuracy of 82% and 88% for a batch size of 64 and 128, respectively	EyePACS
2017, Masood et al. [29]	Transfer learning on CNN based on pre-trained Inception-V3	48.2% accuracy	EyePACS
2018, Kwasigroch et al. [30]	VGG-D architecture with class coding technique	51% accuracy in the assessing stage and 82% in detecting diabetic retinopathy	EyePACS
2018, Wang et al. [1]	AlexNet, VGG16, and Inception-V3	37.43%, 50.03%, 63.23% accuracy, respectively	EyePACS
2018, Garcıa et al. [31]	CNN-based architecture optimized by eliminating noise, performing normalization, and using various hyperparameters	Accuracy of 83.68% with 93.65% specificity	EyePACS
2018, Wan et al. [32]	AlexNet, VGGNet-s, VGGNet-16, VGGNet-19, GoogLeNet, and ResNet after applying transfer learning and hyper-parameter tuning	89.75%, 95.68%, 93.17%, 93.73%, 93.36%, and 90.40% accuracy, respectively	EyePACS
2019, Qummar et al. [6]	Model based on an ensemble of five CNN models including Dense-169, Xception, Dense-121, ResNet-50, and Inception-v3	Precision of 84%, 51%, 65%, 48% and 69% for class 0, 1, 2, 3, and 4, respectively	EyePACS
2019, Shanthi et al. [33]	Alexnet based architecture using suitable rectified linear activation Unit, pooling, and softmax layers	6.6%, 96.2%, 95.6%, and 96.6% accuracy for healthy, stage 1, stage 2, stage 3 cases of diabetic retinopathy, respectively	Messidor
2020, Shankar et al. [34]	Deep learning-based SDL model	99.28% accuracy	Messidor

**Table 2:** Number of images of Messidor-2 corresponding to two different severity grades (SG) of disease.

SG	Messidor-2
0	1593
1	151
Total	1748

**Table 3:** Number of images of EyePACS dataset corresponding five different severity grades (SG) of disease, respectively.

SG	EyePACS
0	25,810
1	2443
2	5292
3	873
4	708
Total	35,126

**Table 4:** Comparison of existing approaches and proposed approach using EyePACS (five categories) and Messidor-2 (two categories) using 10-fold validation. M2 = Messidor-2, EP = EyePACS, I-V3 = Inception-V3, Xp = Xception, RN-50 = ResNet50, M-Net = MobileNet.

Data Sets	VGG16	Xp	M-Net	I-V3	VGG19	RN-50	PA
M2	95.07	93.66	92.59	92.15	87.71	81.99	96
EP	74.66	71.94	74.45	74.61	74.66	74.66	75.09

**Table 5:** Comparison of existing approaches with proposed approach (PA) in terms of % accuracy on the dataset, namely Messidor-2 using 10-fold cross validation.

Dataset	Zago [40]	Gar [41]	Orlando [42]	Voets [43]	Carr [44]	PA
Messidor-2	94.4	94	93.4	85	95	**96**

**Table 6:** Comparison of existing approaches with proposed approach (PA) in terms of % accuracy on dataset, namely EyePACS using 10-fold cross validation.

Dataset	Suriyal [54]	Kaj [55]	Mas [29]	Pratt [18]	Wang [1]	PA
EyePACS	73.30	70.29	48.20	75	63.23	**75.09**

## Supplementary Materials

The Google Colab source codes to replicate the results presented in this paper can be downloaded from this link. https://github.com/kashifyy/ResRF.
